# Neuroprotection by eIF2*α*-CHOP inhibition and XBP-1 activation in EAE/optic neuritiss

**DOI:** 10.1038/cddis.2017.329

**Published:** 2017-07-20

**Authors:** Haoliang Huang, Linqing Miao, Feisi Liang, Xiaodong Liu, Lin Xu, Xiuyin Teng, Qizhao Wang, William H Ridder, Kenneth S Shindler, Yang Sun, Yang Hu

**Affiliations:** 1 Department of Ophthalmology, Stanford University School of Medicine, Palo Alto CA 94304, USA; 2 Shriners Center for Neural Repair and Rehabilitation, Temple University School of Medicine, Philadelphia, PA 19140, USA; 3 Southern California College of Optometry, Marshall B. Ketchum University, Fullerton, CA 92831, USA; 4 Scheie Eye Institute and F.M. Kirby Center for Molecular Ophthalmology, Departments of Ophthalmology and Neurology, University of Pennsylvania, Philadelphia, PA 19104, USA

## Abstract

No therapies exist to prevent neuronal deficits in multiple sclerosis (MS), because the molecular mechanism responsible for the progressive neurodegeneration is unknown. We previously showed that axon injury-induced neuronal endoplasmic reticulum (ER) stress plays an important role in retinal ganglion cell (RGC) death and optic nerve degeneration in traumatic and glaucomatous optic neuropathies. Optic neuritis, one of the most common clinical manifestations of MS, is readily modeled by experimental autoimmune encephalomyelitis (EAE) in mouse. Using this *in vivo* model, we now show that ER stress is induced early in EAE and that modulation of ER stress by inhibition of eIF2*α*-CHOP and activation of XBP-1 in RGC specifically, protects RGC somata and axons and preserves visual function. This finding adds to the evidence that ER stress is a general upstream mechanism for neurodegeneration and suggests that targeting ER stress molecules is a promising therapeutic strategy for neuroprotection in MS.

Multiple sclerosis (MS) is a common inflammatory demyelinating disease and the most frequent chronic neurologic disease of young adults.^1^ Devastating symptoms include dysfunctions in vision, movement and cognition, which are manifested in a relapsing-remitting and/or a chronic-progressive pattern.^1^ Countering the inflammation with immunomodulatory reagents is currently the main therapeutic strategy. This approach effectively reduces inflammation and the severity of acute attacks, but has been much less effective in preventing ongoing neurodegeneration and disability.^2^ An unknown injurious mechanism triggered by inflammatory demyelination of axons may be responsible for axon degeneration, which ultimately leads to retrograde neuronal death and irreversible neurological deficits.^3^ Neuroprotectants have long been sought in MS, but, as in other chronic neurodegenerative diseases, none has been found. The significant clinical need for neuroprotectants compels us to look for the molecular mechanism that mediates neurodegeneration in MS.

Endoplasmic reticulum (ER) stress has emerged as a possible molecular mechanism of neurodegeneration.^4, 5, 6, 7, 8^ When the ER is overwhelmed by unfolded and misfolded proteins, cells experience ER stress and activate a complex cascade of reactions, in general called the unfolded protein response (UPR).^6, 9^ Three ER-resident stress-sensing proteins initiate three evolutionarily conserved UPR pathways: protein kinase RNA-like ER kinase (PERK), inositol-requiring protein-1 (IRE1*α*) and activating transcription factor-6 (ATF6). IRE1*α*, a bi-functional enzyme that contains both a Ser/Thr kinase domain and an endoribonuclease domain, mediates the splicing of X-box binding protein 1 (XBP-1) mRNA to generate an active (spliced) form of the transcription factor, XBP-1s. The IRE1*α*-XBP-1s pathway targets genes that increase ER protein-folding capacity and facilitate degradation of misfolded proteins through ER-associated protein degradation (ERAD). ATF6 is cleaved sequentially by site-1 protease and site-2 protease in Golgi to generate an active transcription factor ATF6 fragment (ATF6f). ATF6f acts alone or forms heterodimers with XBP-1s to induce expression of ER chaperones to promote protein folding and protein degradation through ERAD, which is generally considered cytoprotective.^10, 11^ Interestingly, mutation of ATF6 causes achromatopsia, a cone degeneration disease; and ATF6 deletion mice develop rod and cone dysfunction with aging.^12^ PERK phosphorylates and inactivates eukaryotic translation initiation factor 2*α* (eIF2*α*) to attenuate global mRNA translation and therefore reduce protein load on the ER. However, phosphorylated eIF2*α* (eIF2*α*-P) induces the expression of a pro-apoptotic molecule, C/EBP homologous protein (CHOP), by selectively activating translation of transcription factor ATF4. As a negative feedback mechanism, ATF4 and CHOP induce expression of growth arrest and DNA-damage-inducible protein 34 (GADD34) to facilitate dephosphorylation of eIF2*α*-P and resume global mRNA translation. CHOP mediates ER stress-induced apoptosis through downregulating anti-apoptotic Bcl2, upregulating pro-apoptotic BH-3-only molecules Bim and PUMA,^5^ and increasing expression of death receptor 5 (DR5) and caspase 8 cleavage.^13^ CHOP also can form heterodimers with ATF4 to cause cell death by upregulating protein synthesis and inducing oxidative stress.^14^ Inhibition of CHOP is beneficial in many disease models.^5^ A critical phenomenon of ER stress, which may contribute to the pathogenesis of many disorders, includes the early and transient but protective IRE1*α*-XBP-1 and ATF6 activation *versus* the late and persistent but pro-apoptotic PERK-CHOP activation during chronic ER stress.^15, 16, 17^ Consistent with the theme that sustained activation of the PERK-CHOP pathway in prolonged ER stress causes cell death, previous studies from our laboratory revealed that traumatic injury of the optic nerve (ON) causes sustained CHOP expression, but transient XBP-1s expression, in retinal ganglion cells (RGCs); and that reversal of these processes by deletion of CHOP and activation of XBP-1 synergistically protects RGC somata and axons, and preserves visual function in a mouse glaucoma model.^18, 19^

About 25% of MS patients have optic neuritis as the initial symptom, 70% have it during the course of the disease and about one-third suffer persistent visual symptoms due to ON degeneration and RGC death.^20^ In contrast with other parts of CNS damaged in MS, the pathology and functional deficits of retina/ON injury are more clinically apparent and quantifiable. Therefore multiple clinical trials have used optic neuritis to test neuroprotective therapy.^21^ The rodent experimental autoimmune encephalomyelitis (EAE) model replicates many clinical symptoms and pathological signs of MS, including optic neuritis and significant RGC soma and axon loss.^22, 23^ In the present studies we take advantage of the accessible structures and clear functional readout of the EAE/optic neuritis model, and demonstrate that manipulating these two UPR pathways, PERK-CHOP pathway and IRE1*α*-XBP-1 pathway, provides neuroprotection and preservation of visual function. These results indicate that ER stress is the common upstream signal that triggers the degeneration cascade in both neuronal soma and axon in diverse CNS axonopathies. These insights will help to develop innovative and efficient neuroprotective treatments by encouraging targeting of UPR pathways.

## Results

### The time course of optic neuritis and RGC/ON degeneration in EAE

**Figure 1:**
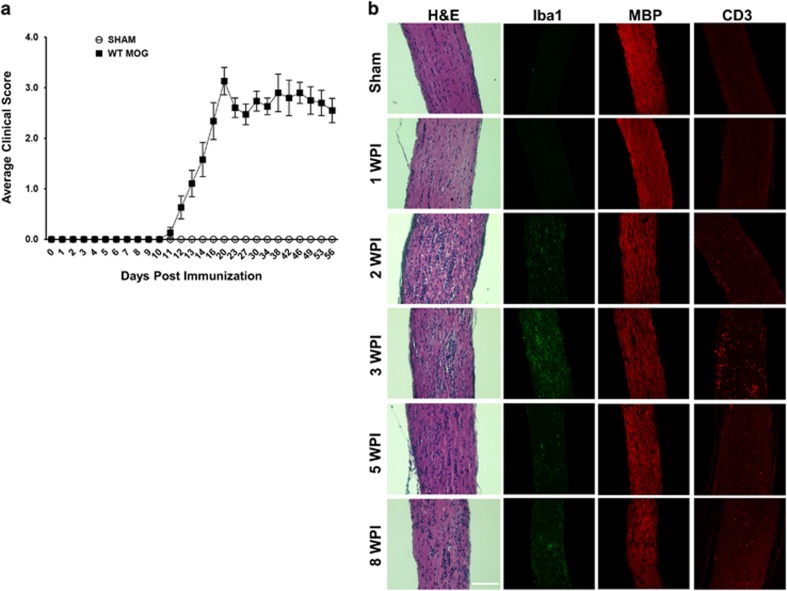
EAE and optic neuritis induced by MOG immunization. (**a**) Time course of EAE score. Data are presented as means±S.E.M and *n*=19. (**b**) ON longitudinal paraffin sections of sham mice and EAE mice at 1, 2, 3, 5 and 8 WPI (weeks post immunization) with H&E staining and immunostaining. Scale bar, 100 *μ*m

**Figure 2:**
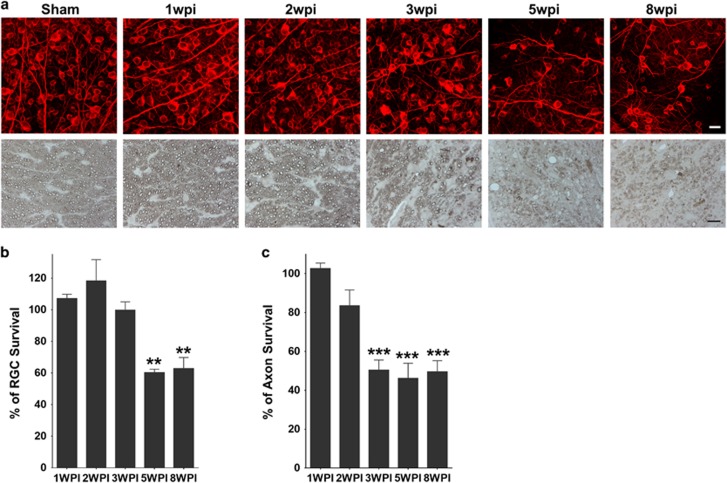
Time course of RGC and ON degeneration in WT EAE mice. (**a**) Upper panel, confocal images of flat-mounted retinas showing surviving Tuj1 positive (red) RGCs at different time points after immunization. Scale bar, 20 *μ*m. Lower panel, light microscope images of semi-thin transverse sections of ON with 1% PPD staining. Scale bar, 10 *μ*m. (**b and c**) Quantification of surviving RGC somata in retina and axons in ON, represented as percentage of EAE mouse eyes compared to the sham control mouse eyes. Data are presented as means±S.E.M., *n*=4, **P<0.01; ***P<0.001; one-way ANOVA with Bonferroni’s *post hoc* test

EAE was induced by immunizing 9-week-old female C57BL/6 mice with myelin oligodendrocyte glycoprotein (MOG). Female mice of this age show a higher incidence of optic neuritis than younger male mice. We observed a distribution of clinical scores that is typical of EAE mice after MOG immunization: the paralysis symptoms started around day 11, peaked around day 20 and plateaued for the next several weeks (Figure 1a). We then examined inflammatory cell infiltration and demyelination in ON at different time points after EAE induction. Optic neuritis began at 2 weeks post immunization (2WPI) and peaked at 3WPI, which is evidenced by the infiltration of inflammatory cells in ON stained with hematoxylin and eosin (H&E) and also recognized by microglial marker Iba1 and T cell marker CD3 (Figure 1b). ON demyelination indicated by diminished MBP staining also peaked at 3WPI (Figure 1b). Significant RGC soma loss was detected later at 5 and 8WPI by quantification of surviving RGC somata in whole-mount retinas (Figures 2a and b). RGC axons are damaged earlier in EAE than RGC somata, as significant axon loss in ON semi-thin cross-sections was already detected at 3WPI by 1% para-phenylenediamine (PPD) staining and continued at 5 and 8WPI (Figures 2a and c). Therefore early inflammation and demyelination in ON may be responsible for later RGC soma and axon degeneration.

### ER stress is induced in RGCs at an early stage of EAE

**Figure 3:**
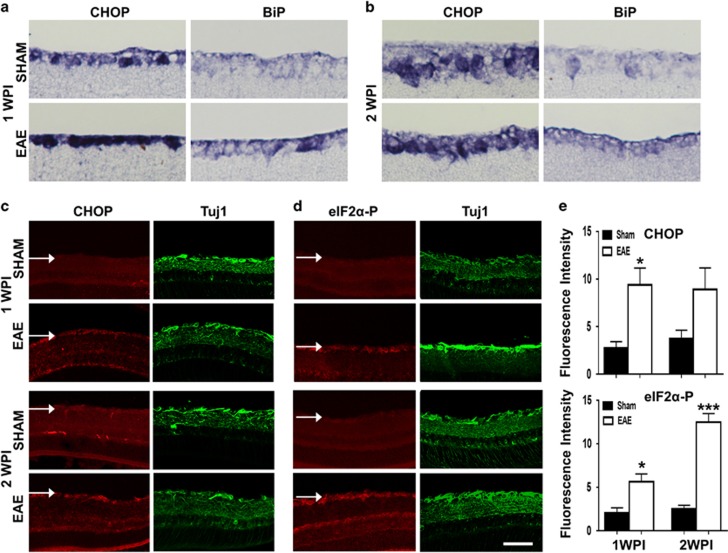
ER stress is induced in RGCs at an early stage of EAE/optic neuritis. (**a and b**) *In situ* hybridization of CHOP and BiP in retina sections of sham and MOG immunized mice at 1 and 2 WPI. (**c and d**) Immunostaining of CHOP, eIF2*α*-P and Tuj1 in retina sections of sham and MOG immunized mice at 1 and 2 WPI. Arrow points to ganglion cell layer (GCL). Scale bar, 100 *μ*m. (**e**) Quantification of fluorescence intensities of CHOP or eIF2*α*-P in Tuj1+ RGCs. Data are presented as means±S.E.M., *n*=4, *P<0.05; ***P<0.001; *T*-test

ER stress has been detected in brain and spinal cord lesions of human MS patients^24^ and of rodents with EAE.^25^ We investigated whether ER stress is also induced in RGCs in EAE mice. *In situ* hybridization revealed increased CHOP and BiP mRNA expression in the ganglion cell layer of retina sections at 1 and 2WPI (Figures 3a and b). Immunohistochemistry confirmed these observations, showing increased levels of CHOP and phosphorylated eIF2*α* (eIF2*α*-P) in Tuj1^+^ RGCs (Figures 3c–e), although the increase of CHOP at 2WPI did not reach statistical significance. We were unable to detect increase of CHOP and eIF2*α*-P in RGCs at later time points (data not shown). These results indicate that EAE induces ER stress in RGCs at early time points, before axon degeneration and neuronal cell death.

### CHOP deletion and XBP-1 activation promote RGC soma and axon survival in EAE

**Figure 4:**
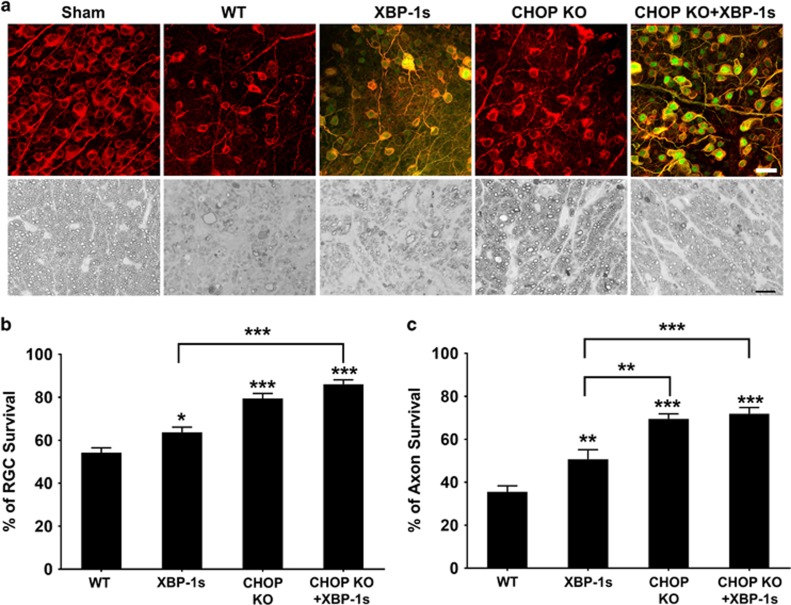
CHOP deletion and XBP-1 activation promote RGC soma and axon survival in EAE mice. (**a**) Upper panel, confocal images of flat-mounted retinas showing surviving Tuj1 positive (red) RGCs at 5 WPI, Scale bar, 20 *μ*m. Lower panel, light microscope images of semi-thin transverse sections of ON with PPD staining at 5 WPI. Scale bar, 10 *μ*m. AAV-XBP-1s-3HA was injected intravitreally 2 weeks before MOG immunization in WT or CHOP KO mice; HA staining (green) shows XBP-1s positive cells. (**b and c**) Quantification of surviving RGC somata and axons at 5 WPI, represented as percentage of EAE mouse eyes compared to the sham control mouse eyes. Data are presented as means±S.E.M and WT, *n*=18; XBP-1s, *n*=14, CHOP KO, *n*=11; CHOP KO+XBP-1s, *n*=11. *P<0.05, **P<0.01; ***P<0.001; one-way ANOVA with Bonferroni’s *post hoc* test

Our previous studies raise the possibility that ER stress is a common neuronal response to disturbances in axonal integrity,^5^ and that the underlying neuronal degeneration mechanism may be similar in inflammatory demyelination-induced axon injury. That EAE induces ER stress in RGCs at early time points suggests that ER stress causes optic neuritis-induced neurodegeneration. To explore this possibility, we asked whether manipulating neuronal intrinsic ER stress in RGCs provides effective neuroprotection in EAE/optic neuritis. We and others have found that adeno-associated virus 2 (AAV2) preferentially infects RGCs and produces sustained transgene expression after intravitreal injection.^26, 27, 28, 29, 30^ Therefore we intravitreously injected AAV2-XBP-1s into WT and CHOP KO mice 2 weeks before MOG immunization and compared these mice with mock-injected mice. Compared to about 54.3% RGC and 35.5% ON survival in WT EAE mice at 5 WPI, RGC and ON survival was significantly increased in CHOP KO mice and in WT mice overexpressing XBP-1s, and further increased to about 86% RGC and 71.9% ON survival in CHOP KO mice with XBP-1s overexpression (Figure 4). WT mice and CHOP KO mice with XBP-1s overexpression showed no significant difference in ON demyelination or Iba1+ inflammatory cells infiltration (Supplementary Figure 1). Therefore, as in glaucoma, manipulation of these two ER stress pathways in opposite directions specifically in RGCs promotes RGC soma and axon survival in EAE/optic neuritis.

### Neuroprotection achieved by blocking CHOP upstream molecule eIF2*α*-P in EAE

**Figure 5:**
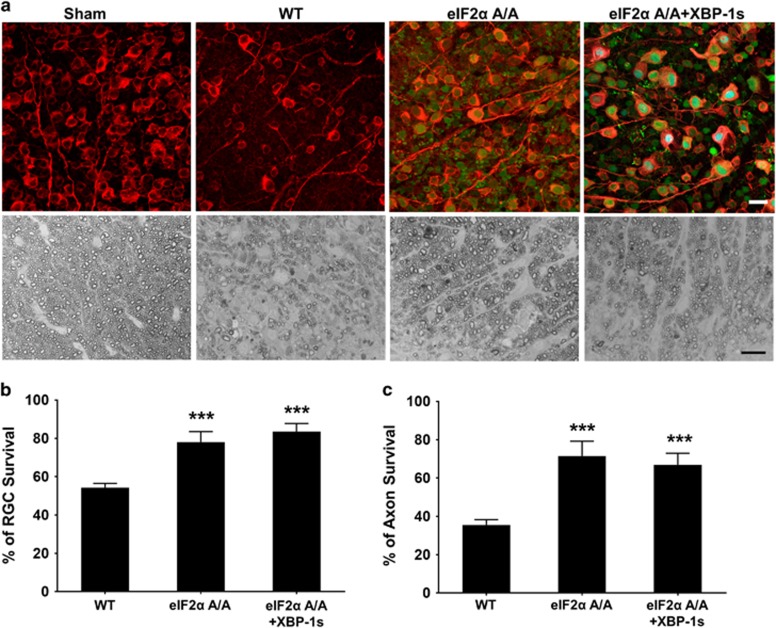
Blocking eIF2*α* phosphorylation promotes neuroprotection of RGC somata and axons in EAE. (**a**) Upper panel, confocal images of flat-mounted retinas showing surviving Tuj1 positive (red) RGC and eIF2*α* deletion/mutant expression in GFP positive cells at 5 WPI. Scale bar, 20 *μ*m. Lower panel, light microscope images of semi-thin transverse sections of ON with PPD staining at 5 WPI. Scale bar, 10 *μ*m. AAV-Cre or AAV-Cre+AAV-XBP-1s was injected intravitreally 2 weeks before MOG immunization in eIF2*α*A/A mice. (**b and c**) Quantification of surviving RGC somata and axons at 5 WPI, represented as percentage of EAE eyes compared to the sham control eyes. Data are presented as means±S.E.M and WT, *n*=18; eIF2*α* A/A, *n*=8; eIF2*α* A/A+XBP-1s, *n*=9. ***P<0.001; one-way ANOVA with Bonferroni’s *post hoc* test

To further confirm that the CHOP pathway plays a detrimental role in EAE/optic neuritis, we conducted a similar analysis to test whether blocking the CHOP upstream regulator eIF2*α*-P specifically in RGCs also provides neuroprotection. We injected AAV2-Cre intravitreally to the eIF2*α* A/A mouse,^31^ which removed the floxed WT eIF2*α* transgene specifically from RGCs and could be identified by its expression of GFP as an indicator, and achieved temporally controlled expression of unphosphorylated eIF2*α* S51A mutant in RGCs. This strategy has been used to confirm that AAV-Cre mediated eIF2*α* S51A mutant expression in RGCs results in CHOP inhibition and protects RGC somata and axons in ON crush and glaucoma models.^19^ Expression of unphosphorylated eIF2*α* S51A mutant in RGCs significantly increased RGC soma and axon survival in EAE (Figure 5), which confirms that inhibiting the eIF2*α*-CHOP pathway provides neuroprotection. Mice with different genetic conditions showed no significant difference in EAE clinical scores (Supplementary Figure 2).

### eIF2*α*-CHOP inhibition and XBP-1 activation also preserve visual function in EAE

**Figure 6:**
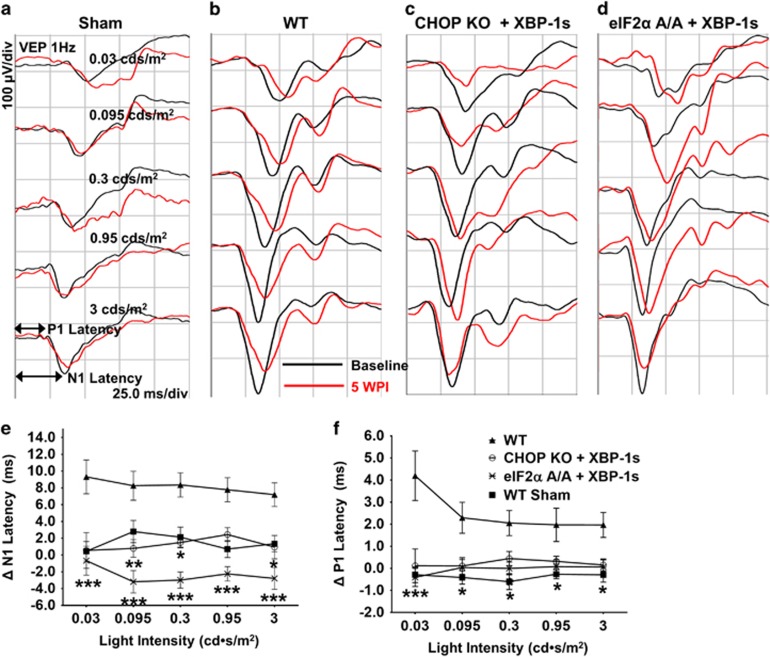
ER stress manipulation preserves visual function of RGC and ON in EAE mice. (**a-d**) Representative waveforms showing the VEP elicited by a series of stimulus light intensities. Black line: baseline, 1 week before MOG immunization; Red line: 5 WPI. P1: the first positive peak after the stimulus flash; N1: the first negative peak after the stimulus flash. (**e and f**) The differences in flash VEP responses between baseline and 5 WPI, represented as ΔN1 and **Δ**P1 latencies with a series of stimulus light intensities. Data are presented as means±S.E.M, WT sham, *n*=8; WT, *n*=23; CHOP KO+XBP-1s, *n*=11; eIF2*α*A/A+XBP-1s, *n*=21. For **Δ**N1, compared to WT, CHOP KO+XBP-1s is significantly different at all light intensities except at 0.95, eIF2*α*A/A+XBP-1s is significantly different at all light intensities. For **Δ**P1, compared to WT, CHOP KO+XBP-1s is significantly different at light intensity 0.03, eIF2*α*A/A+XBP-1s is significantly different at all light intensities, *P<0.05, **P<0.01, ***P<0.001; two-way ANOVA with Bonferroni’s *post hoc* test

The clinical significance of neuroprotection depends on functional preservation. The visual evoked potential (VEP) is a gross electrical signal recorded from visual cortex after flash stimulation of the eye.^32^ Because the VEP signal depends on the integrity of the entire visual pathway, including RGC somata and ON axons, it provides readout of the physiological function of both structures. VEP recording has been used to estimate loss of RGCs and axons in several optic neuropathy models.^33, 34, 35^ Notably, delayed VEP latencies correlate with ON demyelination and axon loss in MS patients.^36, 37^ We recorded the VEP from mice 1 week before MOG immunization (baseline) and at 5 WPI (end point). Because the variation of P1-N1 amplitude was too big to be used in this model, we focused on the more stable N1 and P1 latencies. In contrast to sham mice with constant N1 and P1 latencies (Figure 6a), N1 and P1 latencies were significantly prolonged in WT mice with EAE (Figure 6b); these changes were much smaller in CHOP KO mice injected with AAV-XBP-1s (Figure 6c) and in eIF2*α* A/A mice injected with AAV-Cre+AAV-XBP-1s (Figure 6d). These changes of latencies (Δ N1 or P1=5 WPI–baseline) were significantly different in WT mice and mice with eIF2*α*-CHOP inhibition+XBP-1 activation (Figures 6e and f), indicating that ER stress manipulation preserves visual function.

### Delayed eIF2*α*-CHOP inhibition and XBP-1 activation also provide neuroprotection and preserve visual function in EAE

**Figure 7:**
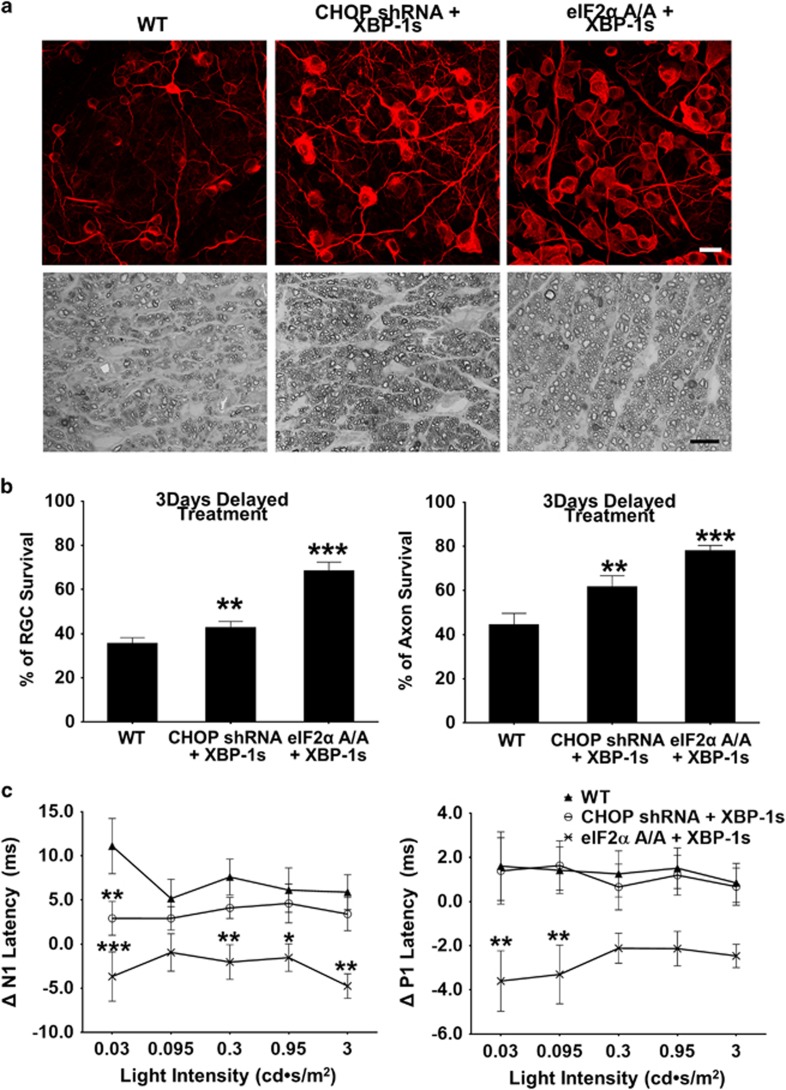
Delayed ER stress manipulation provides neuroprotection and preserves visual function of RGC and ON in EAE mice. (**a**) AAV-CHOP shRNA+AAV-XBP-1s or AAV-Cre+AAV-XBP-1s were injected intravitreally at 3 days post immunization in WT mice and eIF2*α*A/A mice, respectively. Upper panel, confocal images of flat-mounted retinas showing surviving Tuj1 positive (red) RGCs at 8 WPI, Scale bar, 20 *μ*m. Lower panel, light microscope images of semi-thin transverse sections of ON with PPD staining at 8 WPI. Scale bar, 10 *μ*m. (**b**) Quantification of surviving RGC somata and axons, represented as percentage of surviving RGCs or axons in the EAE mouse eyes, compared to the sham control mouse eyes. (**c**) The differences in the flash VEP responses between 1 week before and 7 weeks after MOG immunization are represented as **Δ**N1 latency and **Δ**P1 latency elicited by a series of stimulus light intensities. All quantification data are presented as means±S.E.M., WT, *n*=14; CHOP shRNA+XBP-1s, *n*=14; eIF2*α*A/A+XBP-1s, *n*=10, *P<0.05, **P<0.01, ***P<0.001; two-way ANOVA with Bonferroni’s *post hoc* test

Current immunomodulatory agents effectively reduce inflammation and autoimmune damage in MS, but they fail to prevent progressive neurodegeneration and disability.^2^ For an experimental neuroprotection treatment to be clinically relevant, it must be effective when administered after the onset of disease. To simulate the clinical setting, we tested whether manipulating ER stress only after MOG immunization also protects RGC somata and axons. We have used AAV2-CHOP shRNA to directly knock down CHOP levels in RGCs and demonstrated that it protects RGC somata and axons in ON crush and glaucoma models.^19^ We therefore injected AAV2-CHOP shRNA+AAV2-XBP-1s or AAV2-scramble shRNA+AAV2-GFP (control) into WT mice 3 days after MOG immunization. Because AAV-mediated gene expression in RGCs normally takes at least 2 weeks after infection,^18, 29^ CHOP inhibition and XBP-1 activation are likely to occur 2–3 weeks after MOG immunization. Interestingly, this delayed ER stress modulation significantly protected RGC somata and axons (Figures 7a and b) and significantly decreased the prolonged N1 latency but not P1 latency (Figure 7c). Similarly, eIF2*α* A/A mice injected with AAV-Cre+AAV-XBP-1s 3 days after MOG immunization, also showed RGC soma and axon protection and even better preservation of visual function (Figure 7). Importantly, injecting WT mice with AAV-CHOP shRNA+AAV-XBP-1s 1 week after MOG immunization provided no neuroprotection or visual function preservation (Supplementary Figure 3). These results suggest that the treatment window is limited in the first 2 weeks of EAE in mouse.

## Discussion

By exploiting the anatomical and technical advantages of the optic neuritis mouse model, we confirmed that EAE/optic neuritis induces neuronal ER stress in RGCs. As in traumatic and glaucomatous optic neuropathies, eIF2*α*-CHOP inhibition and XBP-1 activation preserve the morphology and function of both RGC somata and axons after inflammatory demyelination of ON. These results demonstrate that the PERK-eIF2*α*-CHOP branch of UPR in neurons plays an important role in autoimmune-induced neurodegeneration, and that combined inhibition of this pathway and activation of the XBP-1 pathway is a promising therapeutic approach for MS. This study provides additional evidence that neuronal intrinsic ER stress commonly contributes to neurodegeneration and demonstrates the therapeutic potential of targeting neuronal intrinsic UPR pathways in diverse CNS axonopathies.

A previous study found that although EAE induces ER stress in mouse spinal cords, the behavioral rating score did not improve in CHOP KO mice.^38^ Unfortunately, because this study did not examine the spinal cords histologically for neuropathological changes, it lacks morphological evidence concerning neuronal and oligodendrocyte survival in CHOP KO mice with EAE. Indeed, activation of the CHOP upstream molecule PERK in oligodendrocytes is protective in EAE.^39^ The absence of this cell-specific information makes it impossible to interpret why the behavioral deficits did not improve in CHOP KO mice: is CHOP irrelevant or did positive and negative effects of CHOP on neuronal, axonal and oligodendrocyte survival cancel themselves out? Since germ line KO deletes CHOP from all cell populations, the specific cell type that contributes to this phenotype cannot be determined. Our studies using CHOP KO mice also cannot rule out the possibility that CHOP deletion in non-neuronal cells contributes to the protection of RGCs. However, our positive data from eIF2*α* A/A mice and WT mice injected with AAV-CHOP shRNA suggest that neuronal intrinsic inhibition of the eIF2*α*-CHOP pathway is neuroprotective.

The mechanism that induces ER stress in axonopathies is unknown. Release of calcium from intra-axonal smooth ER is an important early event after axon injury that correlates with initiation of axon degeneration,^40, 41^ possibly by promoting cytoskeleton disintegration^42^ and mitochondrial dysfunction.^43^ Of note, disturbance of calcium homeostasis is a major inducer of ER stress.^5^ However, it remains to be determined whether inflammatory demyelination in MS disturbs axonal calcium level.

Modulation of ER stress also protects injured neurons and improves functional recovery in experimental spinal cord injury,^44, 45^ stroke,^46^ Alzheimer disease,^47^ tauopathies,^48^ Parkinson’s disease,^49^ amyotrophic lateral sclerosis,^50, 51^ prion disease^52, 53, 54^ and, most relevantly, retinal cell degenerations, including RGC^18, 19^ and photoreceptor.^12, 16, 55, 56^ Importantly, recent reports provide evidence of ER stress in brain and spinal cord lesions of human MS patients^24^ and rodent EAE,^25^ suggesting the importance of ER stress in the pathophysiology of MS. However, previous studies showed that PERK activation in oligodendrocytes promotes their survival in EAE,^39, 57^ and suggest that ER stress in oligodendrocytes is beneficial in demyelinating diseases.^4, 8^ In contrast to its beneficial role in oligodendrocyte survival, we found that neuronal intrinsic ER stress is injurious to neurons in EAE, as in ON trauma and glaucoma. Therefore, we propose to target the UPR pathways in neurons and oligodendrocytes differently to antagonize the neurodegeneration in MS. Furthermore, the combination of neuroprotective and anti-inflammatory therapeutic strategies may improve the long-term clinical outcome of MS/optic neuritis.

## Materials and Methods

### Animals

CHOP KO and C57BL/6J WT mice were purchased from Jackson Laboratories (Bar Harbor, Maine, USA). eIF2*α* A/A mice were described before.^31^ All mice had a C57BL/6J background. For all surgical and treatment comparisons, control and treatment groups were prepared together in single cohorts, and the experiment repeated at least twice. All experimental procedures were performed in compliance with animal protocols approved by the IACUC at Temple University School of Medicine and APLAC at Stanford University, CA, USA.

### EAE/autoimmune optic neuritis model

Since young mice and male mice are relatively resistant to EAE, we used 8–9-week-old female mice with C57BL/6 background. EAE/optic neuritis was induced by immunization with 150 *μ*g MOG_35-55_ peptide emulsified with complete Freund’s adjuvant (CFA) and 2.5 mg/ml *Mycobacterium tuberculosis*, followed by injection of 200 ng pertussis toxin at day 0 and day 2.^22^ The MOG/CFA mixture was injected subcutaneously at the back of the neck and the base of the tail. Intraperitoneal injection of pertussis toxin in PBS was used to break down the blood-brain barrier. Mice injected with the same volume of CFA emulsion without MOG were used as sham controls. The behavioral deficits of these mice were assessed daily with a 5-point scale:^58^ no disease=0; partial tail paralysis=0.5; tail paralysis or waddling gait=1.0; partial tail paralysis and waddling gait=1.5; tail paralysis and waddling gait=2.0; partial limb paralysis=2.5; paralysis of one limb=3.0; paralysis of one limb and partial paralysis of another=3.5; paralysis of two limbs=4.0; moribund state=4.5; and death=5.0. The clinical score was recorded every day from day 9 to day 14 and twice weekly thereafter until end point.

### Paraffin sections and immunostaining of the eyes

After transcardiac perfusion with 4% paraformaldehyde (PFA) in PBS, the eyes and ONs were dissected out and post-fixed with 4% PFA for 24 h, at 4°C (the lens was extracted after 1 h). The samples were then transferred to sample cassettes, washed in PBS with gentle shaking three times, 10 min each wash, rinsed in 65% ethanol, dehydrated and infiltrated with paraffin in a Shandon Citadel 1000 Tissue Processor (Thermo Fisher Scientific, Waltham, MA, USA). Paraffin sections (9 *μ*m) were cut, rehydrated and incubated with blocking medium (10% goat serum, 0.1% Triton X-100 in PBS) for 1 h at room temperature. Mouse anti-Iba1 (1:200 dilution, Wako, Richmond, VA, USA), rat anti-CD3 (1:100 dilution, Bio-Rad, Hercules, CA, USA), rabbit anti-MBP (1:200 dilution, Covance, Princeton, NJ, USA), FluoroMyelin (1:300 dilution, Thermo Fisher Scientific), mouse anti-CHOP (clone 9C8, 1:200 dilution; Thermo Fisher Scientific), rabbit anti-phospho-eIF2*α* Ser51 (119A11, 1:200 dilution, Cell Signaling, Beverly, MA, USA) and mouse or rabbit neuronal class ß-III tubulin (clone Tuj1, 1:500 dilution; Covance) were diluted in the same blocking buffer. The slides were incubated with primary antibodies overnight at 4 °C, washed three times for 10 min each wash with PBS and incubated in secondary antibodies (Cy2, Cy3 or Cy5-conjugated, 1:200; Jackson ImmunoResearch, West Grove, PA, USA) for 1 h at room temperature. Slides were again washed three times for 10 min each wash with PBS before a cover slip was attached with Fluoromount-G (SouthernBiotech, Birmingham, AL, USA).

### *In situ* hybridization

After mice were perfused with ice-cold 4% PFA/PBS, eyes were dissected out and fixed in 4% PFA/PBS at 4°C overnight. The eyes were dehydrated with increasing concentrations of sucrose solution (15–30%) overnight before embedding in OCT on dry ice. Serial cross sections (12 *μ*m) were cut with a Leica cryostat and collected on Superfrost Plus Slides. The sections were washed twice for 10 min in DEPC-treated PBS and permeabilized twice in 0.1% Tween/PBS for 10 min. After blocking at 50°C for 1 h with hybridization buffer (50% formamide, 5 × SSC, 100 *μ*g/ml torula yeast RNA, 100 *μ*g/ml wheat germ tRNA, 50 *μ*g/ml heparin and 0.1% Tween in DEPC H_2_O), the sections were hybridized with 2 *μ*g biotin-labeled antisense probes at 50°C overnight. The sections were washed three times at 55°C for 10 min with hybridization buffer, 0.1% Tween/PBS, and then blocked in PBS blocking buffer containing 0.1% BSA and 0.2% TritonX-100. The hybridized probes were detected by Streptavidin-AP-conjugate (Roche), and revealed by chromogenic substrate NBT/BCIP (Roche, Basel, Switzerland). Mouse CHOP and BiP probe sequences were from Allen Brain Atlas (http://mouse.brain-map.org/).

### AAV production

The detailed procedure has been described previously.^18, 27^ Briefly, AAV plasmids containing CAG promoter and XBP-1s-3HA, GFP or Cre were co-transfected with pAAV2-RC (Stratagene, La Jolla, CA, USA) and the pHelper plasmid (Stratagene) into HEK293T cells. 72 h after transfection, the cells were lysed to release the viral particles, which were then precipitated by 40% polyethylene glycol and purified by cesium chloride density gradient centrifugation. The fractions with refractive index about 1.371 were taken out for dialysis in MWCO 7000 Slide-A –LYZER cassette (Pierce, Rockford, IL, USA) overnight at 4°C. The AAV titers used for this study were in the range of 1.5-2.5 × 10^12^ genome copy (GC)/ml determined by real-time PCR. Four copies of different mouse CHOP RNAi sequences identified from The RNAi Consortium (5′-ATTTCATCTGAGGACAGGACC-3′ 5′-CATAGAACTCTGACTGGAATC-3′ 5′-TTCCGTTTCCTAGTTCTTCCT-3′ 5′-CGATTTCCTGCTTGAG CCGCT-3′) with modified miR-155 stem-loops and GFP were driven by the uBC promoter in an AAV backbone.^59^

### Intravitreal injection of AAV

The procedures have been described previously.^18, 19, 27, 28^ Briefly, mice were anesthetized by xylazine and ketamine based on their body weight (0.01 mg xylazine/g+0.08 mg ketamine/g). For each AAV intravitreal injection, a micropipette was inserted into the peripheral retina just behind the ora serrata, and advanced into the vitreous chamber so as to avoid damage to the lens. Approximately 2 *μ*l of the vitreous was removed before injection of 2 *μ*l AAV into the vitreous chamber. Eye ointment containing neomycin (Akorn, Somerset, NJ, USA) was applied to protect the cornea after surgery.

### Immunohistochemistry of whole-mount retina and RGC counting

Retinas were dissected out from 4% PFA fixed eyes and washed extensively in PBS before blocking in staining buffer (10% normal goat serum and 2% Triton X-100 in PBS) for half an hour. Mouse neuronal class ß-III tubulin (clone Tuj1, 1:500 dilution; Covance) and rat HA (clone 3F10, 1:200 dilution, Roche,) were diluted in the same staining buffer. Floating retinas were incubated with primary antibodies overnight at 4°C and washed three times for 30 min each with PBS. Secondary antibodies (Cy2, Cy3 or Cy5-conjugated) were then applied (1:200–400; Jackson ImmunoResearch) and incubated for 1 h at room temperature. Retinas were again washed three times for 30 min each with PBS before a cover slip was attached with Fluoromount-G (SouthernBiotech). For RGC counting, whole-mount retinas were immunostained with the TUJ1 antibody and 6–9 fields were randomly sampled from peripheral regions per retina to estimate RGC survival. The investigators who counted the cells were blinded to the treatment of the samples. The percentage of RGC survival was calculated as the ratio of surviving RGC numbers in injured eyes compared to contralateral uninjured eyes.

### Semi-thin sections of ON

After mice were perfused through the heart with ice-cold 4% PFA in PBS, the ON was exposed by removing the brain and post-fixed *in situ* using 2% glutaraldehyde/ 2% PFA in 0.1 M PB for 4 h on ice. Samples were then washed with 0.1 M PB three times, 10 min each wash. The ONs were then carefully dissected out and rinsed with 0.1 M PB three times, 10 min each wash. They were then incubated in 1% osmium tetroxide in 0.1 M PB for 1 h at room temperature followed by washing with 0.1 M PB for 10 min and water for 5 min. ONs were next dehydrated through a series of graded ethanol (50-100%), rinsed twice with propylene oxide (P.O.), 3 min each rinse, and transferred to medium containing 50% EMbed 812 / 50% P.O. overnight. Next day, the medium was changed to a 2:1 ratio of EMbed 812/P.O. ONs remained in this mixture overnight, then were transferred to 100% EMbed 812 on a rotator for another 6 h, embedded in a mold filled with 100% EMbed 812 and incubated at 60 °C overnight. Semi-thin sections (1 *μ*m) were cut on an ultramicrotome (EM UC7, Leica, Wetzlar, Germany) and the transverse sections of ON were collected 1 mm distal to the eye. The semi-thin sections were attached to glass slides and stained with 1% PPD in methanol: isopropanol (1:1) for 35 min. After rinsing three times with methanol: isopropanol (1:1), coverslips were applied with Permount mounting medium (Electron Microscopy Sciences, Hatfield, PA, USA). PPD stains all myelin sheaths, but darkly stains the axoplasm only of degenerating axons, which allows us to differentiate surviving axons from degenerating axons.^60^

### Quantification of surviving axons

The semi-thin sections were viewed through a × 100 lens of a Nikon microscope with a total amplification of × 1000. Sequential images were taken so as to cover the entire area of the ON without overlap. An area of 21.6 *μ*m × 29 *μ*m was cropped from the center of each image, and the surviving axons within the designated area were counted. After counting all the images taken from a single nerve, the mean of the surviving axon number was calculated for each ON. The mean of the surviving axon number in the injured ON was compared to that in the contralateral control ON to yield a percentage of axon survival value. Counts of surviving axons were performed by a single observer who was blinded with regard to the surgical manipulations and treatments.

### Skull screw implant for VEP measurement

7 week-old mice were anesthetized with xylazine and ketamine (0.01 mg xylazine/g+0.08 mg ketamine/g). Sterile ophthalmic ointment (Akorn) was applied to protect the eyes during the procedure. The head was shaved, and a scalp incision was made from the mid line between the ears to 5 mm in front of bregma. After exposing the skull, three small burr holes were drilled with a 0.5 mm micro-drill (Ideal micro-drill, Cellpoint Scientific, Gaithersburg, MD, USA) 2 mm rostral to bregma (reference electrode) and two were drilled 2.5 mm horizontal to lambda (overlying the primary visual cortex, for the active electrodes). Three stainless steel screws (M0.8 X 0.120 SL FILLSS. PN: NAS721CE80-120, Antrin Miniature Specialties INC, Fallbrook, CA, USA) were mounted in the holes and sealed with dental cement, 0.5 mm of their tips were inside the skull. The scalp was closed with surgical sutures to cover the edge of the cement. Antibiotic ointment was applied to the wound after surgery to prevent contamination. Mice recovered for 1 week before the first VEP measurement.

### VEP measurements

Mice were anesthetized with xylazine and ketamine. The pupils were dilated by applying 1% tropicamide sterile ophthalmic solution (Akorn). One drop of an artificial tear (Alcon, Fort Worth, TX, USA) was applied regularly to the eyes to keep the corneas moist and prevent cataract formation throughout the procedure. The mice were kept in the dark for 5–10 min before the measurement. Therefore, the VEP measurement always began 25 min after the mice were anesthetized. The right eye was examined first; the left eye was occluded with black tape. One alligator clip was attached to the active electrode over the left visual cortex and another to the reference electrode. Mice were placed in the ganzfeld of the VEP instrument (RETI-port, Roland Consult, Havel, Germany) in a dark room; body temperature was maintained at 37 °C using a feedback-controlled heating pad (Physitemp Instruments Inc., Clifton, NJ, USA). The VEP was recorded in response to a series of white light flashes of graded intensity from 0.030 cds/m^2^ to 3.0 cds/m^2^. The flashes were presented at a frequency of 1 per second (1 Hz) and 30 flashes were averaged for a response. After recording from the right eye was complete, the black tape was removed from the left eye and placed over the right eye, and the alligator clip was moved from the left to the right side of the visual cortex. The VEP of the left eye was recorded under the same conditions as the right eye. After recording, the black tape was removed and antibiotic ointment (Akorn) applied to the eyes. The first positive peak in the VEP waveform was designated as P1 and the first negative peak as N1. The latency of P1 or N1 and the P1-N1 amplitude were measured one week before MOG immunization as a baseline and at the end point of 5WPI. The differences between the baseline and end point recordings under different stimulation intensities were calculated and referred to as Δ P1 latency, Δ N1 latency and Δ P1-N1 amplitude.

### Statistics

Data are presented as means±S.E.M. We used Student’s *t*-test for two-group comparison, one-way ANOVA with Bonferroni’s *post hoc* test for multiple comparisons (≥3 groups) with one variance and two-way ANOVA with Bonferroni’s *post hoc* test for multiple comparisons (≥3 groups) with two variances.

## References

[bib1] Frohman EM, Racke MK, Raine CS. Multiple sclerosis—the plaque and its pathogenesis. N Engl J Med 2006; 354: 942–955. 16510748 10.1056/NEJMra052130

[bib2] Ontaneda D, Fox RJ, Chataway J. Clinical trials in progressive multiple sclerosis: lessons learned and future perspectives. Lancet Neurol 2015; 14: 208–223. 25772899 10.1016/S1474-4422(14)70264-9 PMC4361791

[bib3] Traka M, Podojil JR, McCarthy DP, Miller SD, Popko B. Oligodendrocyte death results in immune-mediated CNS demyelination. Nat Neurosci 2016; 19: 65–74. 26656646 10.1038/nn.4193 PMC4837900

[bib4] Lin W, Popko B. Endoplasmic reticulum stress in disorders of myelinating cells. Nat Neurosci 2009; 12: 379–385. 19287390 10.1038/nn.2273 PMC2697061

[bib5] Li S, Yang L, Selzer ME, Hu Y. Neuronal endoplasmic reticulum stress in axon injury and neurodegeneration. Ann Neurol 2013; 74: 768–777. 23955583 10.1002/ana.24005 PMC3963272

[bib6] Wang M, Kaufman RJ. Protein misfolding in the endoplasmic reticulum as a conduit to human disease. Nature 2016; 529: 326–335. 26791723 10.1038/nature17041

[bib7] Smith HL, Mallucci GR. The unfolded protein response: mechanisms and therapy of neurodegeneration. Brain 2016; 139: 2113–2121. 27190028 10.1093/brain/aww101 PMC4958893

[bib8] Way SW, Popko B. Harnessing the integrated stress response for the treatment of multiple sclerosis. Lancet Neurol 2016; 15: 434–443. 26873788 10.1016/S1474-4422(15)00381-6 PMC4792730

[bib9] Walter P, Ron D. The unfolded protein response: from stress pathway to homeostatic regulation. Science 2011; 334: 1081–1086. 22116877 10.1126/science.1209038

[bib10] Lynch JM, Maillet M, Vanhoutte D, Schloemer A, Sargent MA, Blair NS et al. A thrombospondin-dependent pathway for a protective ER stress response. Cell 2012; 149: 1257–1268. 22682248 10.1016/j.cell.2012.03.050 PMC3372931

[bib11] Wu J, Rutkowski DT, Dubois M, Swathirajan J, Saunders T, Wang J et al. ATF6 alpha optimizes long-term endoplasmic reticulum function to protect cells from chronic stress. Dev Cell 2007; 13: 351–364. 17765679 10.1016/j.devcel.2007.07.005

[bib12] Kohl S, Zobor D, Chiang WC, Weisschuh N, Staller J, Gonzalez Menendez I et al. Mutations in the unfolded protein response regulator ATF6 cause the cone dysfunction disorder achromatopsia. Nat Genet 2015; 47: 757–765. 26029869 10.1038/ng.3319 PMC4610820

[bib13] Lu M, Lawrence DA, Marsters S, Acosta-Alvear D, Kimmig P, Mendez AS et al. Opposing unfolded-protein-response signals converge on death receptor 5 to control apoptosis. Science 2014; 345: 98–101. 24994655 10.1126/science.1254312 PMC4284148

[bib14] Han J, Back SH, Hur J, Lin YH, Gildersleeve R, Shan J et al. ER-stress-induced transcriptional regulation increases protein synthesis leading to cell death. Nat Cell Biol 2013; 15: 481–490. 23624402 10.1038/ncb2738 PMC3692270

[bib15] Rutkowski DT, Arnold SM, Miller CN, Wu J, Li J, Gunnison KM et al. Adaptation to ER stress is mediated by differential stabilities of pro-survival and pro-apoptotic mRNAs and proteins. PLoS Biol 2006; 4: e374. 17090218 10.1371/journal.pbio.0040374 PMC1634883

[bib16] Lin JH, Li H, Yasumura D, Cohen HR, Zhang C, Panning B et al. IRE1 signaling affects cell fate during the unfolded protein response. Science 2007; 318: 944–949. 17991856 10.1126/science.1146361 PMC3670588

[bib17] Lin JH, Li H, Zhang Y, Ron D, Walter P. Divergent effects of PERK and IRE1 signaling on cell viability. PLoS ONE 2009; 4: e4170. 19137072 10.1371/journal.pone.0004170 PMC2614882

[bib18] Hu Y, Park KK, Yang L, Wei X, Yang Q, Cho KS et al. Differential effects of unfolded protein response pathways on axon injury-induced death of retinal ganglion cells. Neuron 2012; 73: 445–452. 22325198 10.1016/j.neuron.2011.11.026 PMC3278720

[bib19] Yang L, Li S, Miao L, Huang H, Liang F, Teng X et al. Rescue of glaucomatous neurodegeneration by differentially modulating neuronal endoplasmic reticulum stress molecules. J Neurosci 2016; 36: 5891–5903. 27225776 10.1523/JNEUROSCI.3709-15.2016 PMC4879204

[bib20] Balcer LJ, Miller DH, Reingold SC, Cohen JA. Vision and vision-related outcome measures in multiple sclerosis. Brain 2015; 138(Pt 1): 11–27. 25433914 10.1093/brain/awu335 PMC4285195

[bib21] Aktas O, Albrecht P, Hartung HP. Optic neuritis as a phase 2 paradigm for neuroprotection therapies of multiple sclerosis: update on current trials and perspectives. Curr Opin Neurol 2016; 29: 199–204. 27035900 10.1097/WCO.0000000000000327

[bib22] Quinn TA, Dutt M, Shindler KS. Optic neuritis and retinal ganglion cell loss in a chronic murine model of multiple sclerosis. Front Neurol 2011; 2: 50. 21852980 10.3389/fneur.2011.00050 PMC3151613

[bib23] Fonseca-Kelly Z, Nassrallah M, Uribe J, Khan RS, Dine K, Dutt M et al. Resveratrol neuroprotection in a chronic mouse model of multiple sclerosis. Front Neurol 2012; 3: 84. 22654783 10.3389/fneur.2012.00084 PMC3359579

[bib24] McMahon J, McQuaid S, Reynolds R, Fitzgerald U. Increased expression of ER stress- and hypoxia-associated molecules in grey matter lesions in multiple sclerosis. Mult Scler 2012; 18: 1437–1447. 22354737 10.1177/1352458512438455

[bib25] Ni Fhlathartaigh M, McMahon J, Reynolds R, Connolly D, Higgins E, Counihan T et al. Calreticulin and other components of endoplasmic reticulum stress in rat and human inflammatory demyelination. Acta Neuropathol Commun 2013; 1: 37. 24252779 10.1186/2051-5960-1-37 PMC3893522

[bib26] Park KK, Liu K, Hu Y, Smith PD, Wang C, Cai B et al. Promoting axon regeneration in the adult CNS by modulation of the PTEN/mTOR pathway. Science 2008; 322: 963–966. 18988856 10.1126/science.1161566 PMC2652400

[bib27] Yang L, Miao L, Liang F, Huang H, Teng X, Li S et al. The mTORC1 effectors S6K1 and 4E-BP play different roles in CNS axon regeneration. Nat Commun 2014; 5: 5416. 25382660 10.1038/ncomms6416 PMC4228696

[bib28] Miao L, Yang L, Huang H, Liang F, Ling C, Hu Y. mTORC1 is necessary but mTORC2 and GSK3beta are inhibitory for AKT3-induced axon regeneration in the central nervous system. eLife 2016; 5: e14908. 27026523 10.7554/eLife.14908 PMC4841781

[bib29] Boye SE, Boye SL, Lewin AS, Hauswirth WW. A comprehensive review of retinal gene therapy. Mol Ther 2013; 21: 509–519. 23358189 10.1038/mt.2012.280 PMC3642288

[bib30] Pang JJ, Lauramore A, Deng WT, Li Q, Doyle TJ, Chiodo V et al. Comparative analysis of *in vivo* and *in vitro* AAV vector transduction in the neonatal mouse retina: effects of serotype and site of administration. Vision Res 2008; 48: 377–385. 17950399 10.1016/j.visres.2007.08.009

[bib31] Back SH, Scheuner D, Han J, Song B, Ribick M, Wang J et al. Translation attenuation through eIF2alpha phosphorylation prevents oxidative stress and maintains the differentiated state in beta cells. Cell Metab 2009; 10: 13–26. 19583950 10.1016/j.cmet.2009.06.002 PMC2742645

[bib32] Ridder WH 3rd, Nusinowitz S. The visual evoked potential in the mouse—origins and response characteristics. Vision Res 2006; 46: 902–913. 16242750 10.1016/j.visres.2005.09.006

[bib33] Heiduschka P, Schnichels S, Fuhrmann N, Hofmeister S, Schraermeyer U, Wissinger B et al. Electrophysiological and histologic assessment of retinal ganglion cell fate in a mouse model for OPA1-associated autosomal dominant optic atrophy. Invest Ophthalmol Vis Sci 2010; 51: 1424–1431. 19834041 10.1167/iovs.09-3606

[bib34] Sullivan TA, Geisert EE, Hines-Beard J, Rex TS. Systemic adeno-associated virus-mediated gene therapy preserves retinal ganglion cells and visual function in DBA/2J glaucomatous mice. Hum Gene Ther 2011; 22: 1191–1200. 21542676 10.1089/hum.2011.052 PMC3205793

[bib35] You Y, Klistorner A, Thie J, Graham SL. Latency delay of visual evoked potential is a real measurement of demyelination in a rat model of optic neuritis. Invest Ophthalmol Vis Sci 2011; 52: 6911–6918. 21791585 10.1167/iovs.11-7434

[bib36] Klistorner A, Arvind H, Nguyen T, Garrick R, Paine M, Graham S et al. Axonal loss and myelin in early ON loss in postacute optic neuritis. Ann Neurol 2008; 64: 325–331. 18825673 10.1002/ana.21474

[bib37] Diem R, Tschirne A, Bahr M. Decreased amplitudes in multiple sclerosis patients with normal visual acuity: a VEP study. J Clin Neurosci 2003; 10: 67–70. 12464525 10.1016/s0967-5868(02)00172-8

[bib38] Deslauriers AM, Afkhami-Goli A, Paul AM, Bhat RK, Acharjee S, Ellestad KK et al. Neuroinflammation and endoplasmic reticulum stress are coregulated by crocin to prevent demyelination and neurodegeneration. J Immunol 2011; 187: 4788–4799. 21964030 10.4049/jimmunol.1004111

[bib39] Lin W, Lin Y, Li J, Fenstermaker AG, Way SW, Clayton B et al. Oligodendrocyte-specific activation of PERK signaling protects mice against experimental autoimmune encephalomyelitis. J Neurosci 2013; 33: 5980–5991. 23554479 10.1523/JNEUROSCI.1636-12.2013 PMC3654380

[bib40] Wang JT, Medress ZA, Barres BA. Axon degeneration: molecular mechanisms of a self-destruction pathway. J Cell Biol 2012; 196: 7–18. 22232700 10.1083/jcb.201108111 PMC3255986

[bib41] Howell GR, Soto I, Libby RT, John SW. Intrinsic axonal degeneration pathways are critical for glaucomatous damage. Exp Neurol 2013; 246: 54–61. 22285251 10.1016/j.expneurol.2012.01.014 PMC3831512

[bib42] Ma M, Ferguson TA, Schoch KM, Li J, Qian Y, Shofer FS et al. Calpains mediate axonal cytoskeleton disintegration during Wallerian degeneration. Neurobiol Dis 2013; 56: 34–46. 23542511 10.1016/j.nbd.2013.03.009 PMC3721029

[bib43] Villegas R, Martinez NW, Lillo J, Pihan P, Hernandez D, Twiss JL et al. Calcium release from intra-axonal endoplasmic reticulum leads to axon degeneration through mitochondrial dysfunction. J Neurosci 2014; 34: 7179–7189. 24849352 10.1523/JNEUROSCI.4784-13.2014 PMC4028495

[bib44] Penas C, Font-Nieves M, Fores J, Petegnief V, Planas A, Navarro X et al. Autophagy, and BiP level decrease are early key events in retrograde degeneration of motoneurons. Cell Death Differ 2011; 18: 1617–1627. 21436843 10.1038/cdd.2011.24 PMC3172115

[bib45] Valenzuela V, Collyer E, Armentano D, Parsons GB, Court FA, Hetz C. Activation of the unfolded protein response enhances motor recovery after spinal cord injury. Cell Death Dis 2012; 3: e272. 22337234 10.1038/cddis.2012.8 PMC3288350

[bib46] Krajewska M, Xu L, Xu W, Krajewski S, Kress CL, Cui J et al. Endoplasmic reticulum protein BI-1 modulates unfolded protein response signaling and protects against stroke and traumatic brain injury. Brain Res 2011; 1370: 227–237. 21075086 10.1016/j.brainres.2010.11.015 PMC3019258

[bib47] Ma T, Trinh MA, Wexler AJ, Bourbon C, Gatti E, Pierre P et al. Suppression of eIF2alpha kinases alleviates Alzheimer's disease-related plasticity and memory deficits. Nat Neurosci 2013; 16: 1299–1305. 23933749 10.1038/nn.3486 PMC3756900

[bib48] Radford H, Moreno JA, Verity N, Halliday M, Mallucci GR. PERK inhibition prevents tau-mediated neurodegeneration in a mouse model of frontotemporal dementia. Acta Neuropathol 2015; 130: 633–642. 26450683 10.1007/s00401-015-1487-z PMC4612323

[bib49] Valdes P, Mercado G, Vidal RL, Molina C, Parsons G, Court FA et al. Control of dopaminergic neuron survival by the unfolded protein response transcription factor XBP1. Proc Natl Acad Sci USA 2014; 111: 6804–6809. 24753614 10.1073/pnas.1321845111 PMC4020088

[bib50] Kim HJ, Raphael AR, LaDow ES, McGurk L, Weber RA, Trojanowski JQ et al. Therapeutic modulation of eIF2alpha phosphorylation rescues TDP-43 toxicity in amyotrophic lateral sclerosis disease models. Nat Genet 2014; 46: 152–160. 24336168 10.1038/ng.2853 PMC3934366

[bib51] Saxena S, Cabuy E, Caroni P. A role for motoneuron subtype-selective ER stress in disease manifestations of FALS mice. Nat Neurosci 2009; 12: 627–636. 19330001 10.1038/nn.2297

[bib52] Moreno JA, Halliday M, Molloy C, Radford H, Verity N, Axten JM et al. Oral treatment targeting the unfolded protein response prevents neurodegeneration and clinical disease in prion-infected mice. Sci Transl Med 2013; 5: 206ra138. 10.1126/scitranslmed.3006767 24107777

[bib53] Moreno JA, Radford H, Peretti D, Steinert JR, Verity N, Martin MG et al. Sustained translational repression by eIF2alpha-P mediates prion neurodegeneration. Nature 2012; 485: 507–511. 22622579 10.1038/nature11058 PMC3378208

[bib54] Halliday M, Radford H, Sekine Y, Moreno J, Verity N, le Quesne J et al. Partial restoration of protein synthesis rates by the small molecule ISRIB prevents neurodegeneration without pancreatic toxicity. Cell Death Dis 2015; 6: e1672. 25741597 10.1038/cddis.2015.49 PMC4385927

[bib55] Chan P, Stolz J, Kohl S, Chiang WC, Lin JH. Endoplasmic reticulum stress in human photoreceptor diseases. Brain Res 2016; 1648(Pt B): 538–541. 27117871 10.1016/j.brainres.2016.04.021 PMC5036988

[bib56] Ghosh R, Wang L, Wang ES, Perera BG, Igbaria A, Morita S et al. Allosteric inhibition of the IRE1alpha RNase preserves cell viability and function during endoplasmic reticulum stress. Cell 2014; 158: 534–548. 25018104 10.1016/j.cell.2014.07.002 PMC4244221

[bib57] Hussien Y, Cavener DR, Popko B. Genetic inactivation of PERK signaling in mouse oligodendrocytes: normal developmental myelination with increased susceptibility to inflammatory demyelination. Glia 2014; 62: 680–691. 24481666 10.1002/glia.22634 PMC6342275

[bib58] Gran B, Zhang GX, Yu S, Li J, Chen XH, Ventura ES et al. IL-12p35-deficient mice are susceptible to experimental autoimmune encephalomyelitis: evidence for redundancy in the IL-12 system in the induction of central nervous system autoimmune demyelination. J Immunol 2002; 169: 7104–7110. 12471147 10.4049/jimmunol.169.12.7104

[bib59] Chung KH, Hart CC, Al-Bassam S, Avery A, Taylor J, Patel PD et al. Polycistronic RNA polymerase II expression vectors for RNA interference based on BIC/miR-155. Nucleic Acids Res 2006; 34: e53. 16614444 10.1093/nar/gkl143 PMC1435982

[bib60] Smith RS Systematic evaluation of the mouse eye: anatomy, pathology, and biomethods. CRC Press: Boca Raton, FL, USA, 2002.

