# Supplementary material for: Neuroprotection by eIF2α-CHOP inhibition and XBP-1 activation in EAE/optic neuritiss
Source: Cell Death Dis. 2017 Jul 20;8(7):e2936–. doi: 10.1038/cddis.2017.329 (PMC5550873; doi:10.1038/cddis.2017.329)
Supplement: Supplementary Figures [file cddis2017329x1.doc]

**Supplemental Information**


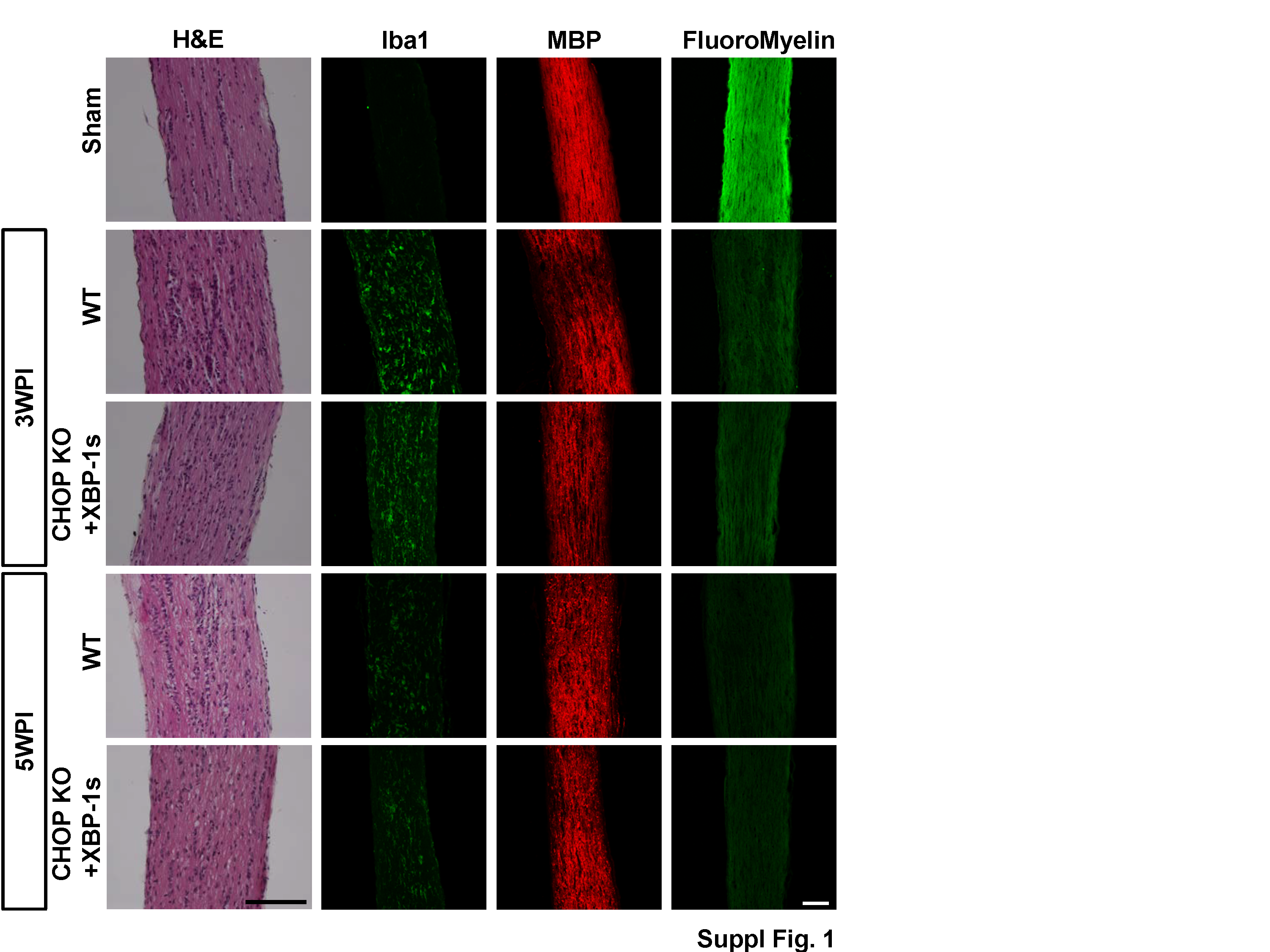


**Figure S1. ON inflammation is not affected by CHOP deletion and XBP-1 activation.**

ON longitudinal paraffin sections of sham mice and EAE mice at 3 and 5 WPI with H&E staining and immunostaining. Scale bar, 100µm.

**

**

**Figure S2. Comparable EAE scores of mice with different genetic conditions.**

Time course of EAE scores in mice with different genetic backgrounds. Data are presented as means ± s.e.m and n=10-19.

**
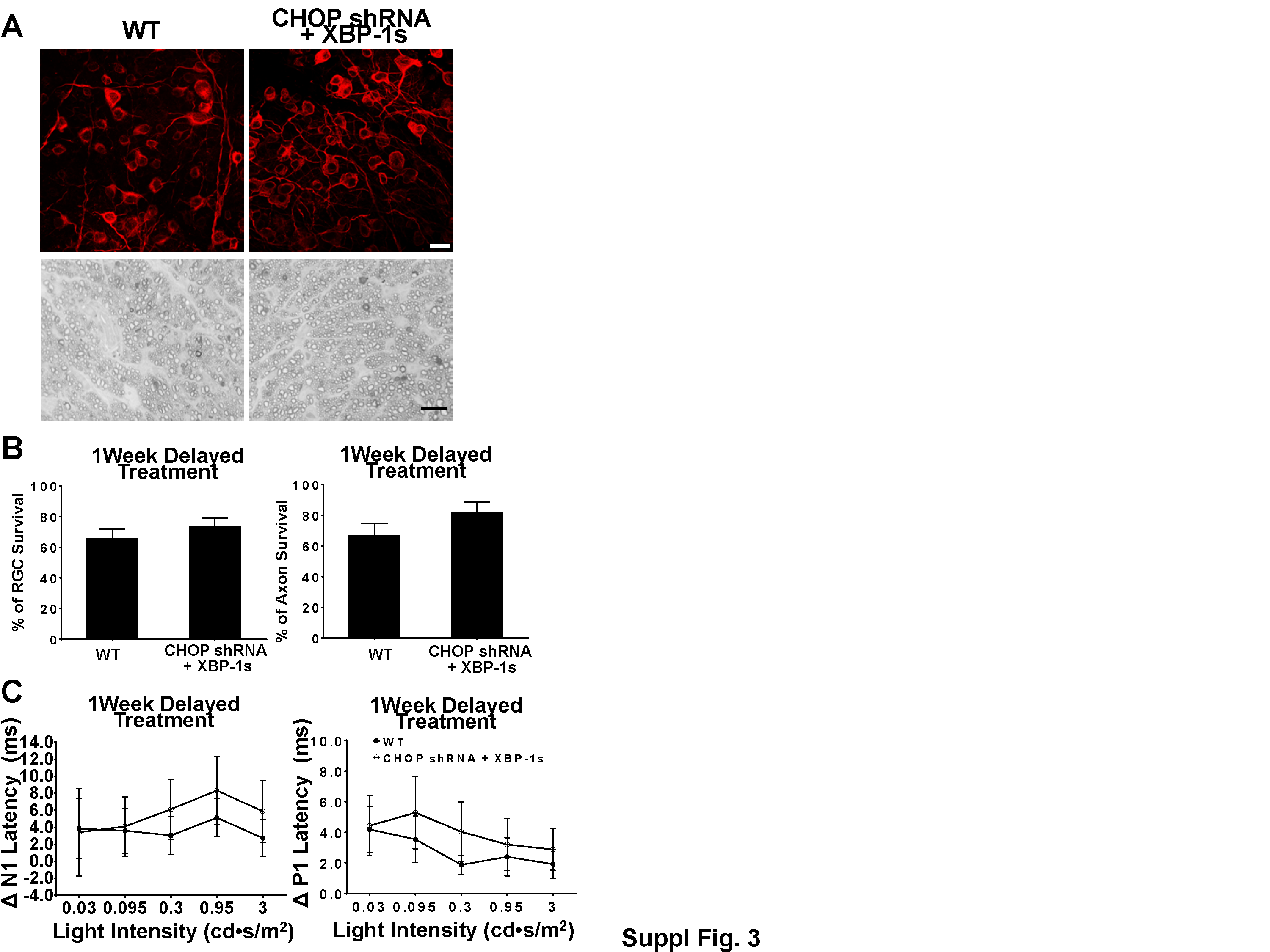
**

**Figure S3. No neuroprotection by further delayed ER stress manipulation in EAE mice.**

**(A)** AAVs were injected one week after MOG injection. Confocal images of flat-mounted retinas showing surviving Tuj1 positive RGCs in upper panel; light microscope images of semi-thin transverse sections of ON at 1 mm distal to the eye with PPD staining in lower panel at 8 WPI. Scale bar, 20 µm in upper panel, 10 µm in lower panel. **(B)** Quantification of surviving RGCs in retina and axons in ON, represented as percentage of surviving RGCs or axons in the EAE mouse eyes, compared to the sham control mouse eyes. Data are presented as means ± s.e.m, n=11. **(C)** The differences in flash VEP responses between 1 week before and 7 weeks after MOG injection are represented as ΔN1 latency and ΔP1 latency using a series of stimulus light intensities. Data are presented as means ± s.e.m, n=11.
